# Intramedullary cortical bone strut improves the cyclic stability of osteoporotic proximal humeral fractures

**DOI:** 10.1186/s12891-017-1421-8

**Published:** 2017-02-02

**Authors:** Chih-Kun Hsiao, Yi-Jung Tsai, Cheng-Yo Yen, Cheng-Hung Lee, Teng-Yao Yang, Yuan-Kun Tu

**Affiliations:** 1 Department of Medical Research, E-Da Hospital, No. 1 E-Da Rd, Yuan-Chau District, Kaohsiung, Taiwan; 2 Department of Orthopedics, E-Da Hospital, No. 1 E-Da Rd, Yuan-Chau District, Kaohsiung, Taiwan; 3 Department of Orthopedics, Veterans General Hospital, No.1650 Taiwan Boulevard Sect. 4, Taichung, Taiwan

**Keywords:** Osteoporosis, Humeral fractures, Locking plate, Intramedullary strut, Stiffness, Cyclic loading

## Abstract

**Background:**

Proximal humeral fractures treated with locking plate can fail due to varus collapse, especially in osteoporotic bone with medial cortex comminution. The use of an intramedullary strut together with locking plate fixation may strengthen fixation and provide additional medial support to prevent the varus malalignment. This study biomechanically investigates the influence of an intramedullary cortical bone strut on the cyclic stability of proximal humeral fractures stabilized by locking plate fixation in a cadaver model.

**Methods:**

Ten cadaveric humeri were divided into two groups statistically matched for bone density. Each specimen was osteotomized with 10 mm gap at the surgical neck. The non-augmented group stabilized with locking plate alone; in the augmented group, a locking plate was used combined with an intramedullary cortical bone strut. The strut was retrograded into the subchondral bone, and three humeral head screws were inserted into the strut to form a plate-screw-strut mechanism. The cyclic axial load was performed to 450 N for 6000 cycles and then loaded to failure. Construct stiffness, cyclic loading behavior and failure strength were analyzed to identify differences between groups.

**Results:**

The augmented constructs were significantly stiffer than the non-augmented constructs during cycling. On average, the maximum displacements at 6000 cycles for non-augmented and augmented groups were 3.10 ± 0.75 mm and 1.7 ± 0.65 mm (*p* = 0.01), respectively. The mean peak-to-peak (inter cycle) displacement at 6000 cycles was about 2 times lower for the augmented group (1.36 ± 0.68 mm vs. 2.86 ± 0.51 mm). All specimens showed varus collapse combined with loss of screw fixation of the humeral head. The failure load of the augmented group was increased by 2.0 (SD = 0.41) times compared with the non-augmented group (*p* < 0.001).

**Conclusions:**

The stability and strength of the locking plate augmented with an intramedullary strut were significantly increased. For bone with poor quality, the subsidence of the locked screws led larger displacement, decreased the stability of the constructs, however, the plate-screw-strut mechanism provided more rigidity to stabilize the fixation. This study emphasized the importance of intramedullary support for the proximal humeral fractures fixed with a locked plate under cyclic loading, especially in bone with poor quality. This work is based on the results of cadaver model, further in vivo analysis is necessary to determine if the clinical results can be extrapolated from this data.

## Background

Proximal humerus fractures are the most common fractures in the elderly and account for approximately 5% of all fractures [1–3]. They can be successfully managed by various surgical methods, including open reduction, stabilization using plates and screws, interlocking nails, or an external fixator in patients with healthy bone. Nevertheless, the stability of proximal humeral fractures remains difficult if osteoporosis or severe loss of bone stock is present. Biomechanical studies have shown that locking plates are significantly beneficial in cases of comminuted proximal humerus fractures, and that they demonstrate potential in providing greater stiffness during cycling and failure strength than traditional compression fixation techniques [4–7]. Thus, locking plate systems has become one of the most popular techniques to treat proximal humerus fractures.

Clinical studies, however, have shown variable results of locked plate fixation of proximal humerus fractures, and complications such as intra-articular screw penetration or varus collapse of the fracture, especially in osteoporotic bone or in fractures with medial metaphyseal comminution have been reported [8–17]. It has been reported that these complications are caused by the locking plates being placed on the lateral proximal humerus without medial column support [18, 19], and recent studies have emphasized the importance of mechanical support of the medial column to reduce these complications [4, 20, 21]. Biomechanical testing has also shown that the intramedullary fibular allografts combined with locking plate fixation can provide medial support, increase the overall stiffness of the construct, and reduce migration of the humeral head fragment compared with a locking plate alone [12, 13, 17, 20, 22–24]. Although many biomechanical studies have focused on the static stability and failure strength of constructs, few have discussed the cyclic stability of humerus fractures treated with a locking plate and intramedullary support, especially for patients with osteoporosis. Therefore, the potential effect of intramedullary struts on the dynamic behavior of osteoporotic humerus fractures remains unclear.

The objective of this study was to investigate the influence of an intramedullary strut on the biomechanical properties of osteoporotic proximal humeral fractures stabilized by locking plate fixation in a cadaver model. We hypothesized that in the case of osteoporosis, a locking plate augmented with an intramedullary cortical strut would provide superior cyclic stability and post-cyclic failure strength compared with fixation using locking plate alone.

## Methods

### Preparation of specimens

Ten freshly frozen proximal humeri (average age 75.4 years; range 64–88 years; 4 males and 6 females) were acquired from the Anatomic Gift Foundation, Inc. (Hanover, Germany). The usage of cadaver specimens in this study was approved by the institutional review board in E-Da Hospital (EMRP11098N RII). All specimens were divided into two groups of five specimens each, and the bone densities in each group were matched as far as possible to minimize variations in bone quality. Table 1 shows the gender, age, and average bone mineral density (BMD) data for the two groups. Both groups were statistically similar with regards to age (*p* = 0.472). To identify similarities in the bone properties between groups, the bone mineral density (BMD) was evaluated to compare their differences. The BMD of the specimens was determined using computed tomography (CT) scans evaluating a circular area of transverse sectional images at the level of maximum diameter of the humeral head [25, 26]. The relative values of bone densities were obtained using two known density calibration phantoms (160 and 320 mg/cm^3^) scanned simultaneously with the humerus. In Table 1, the mean BMD for the non-augmented group was statistically similar to the augmented group (non-augmented group, 247.4 ± 50.8 mg/cm^3^ vs. augmented group, 244.4 ± 36.3 mg/cm^3^; *p* = 0.917). Before the experiments, each specimen was gradually warmed to room temperature (22 ± 2 °C) until fully thawed (about 10 h). All soft tissues were removed and the mid-shaft was cut 18 cm from the top of the humeral head, and then the distal portion of the shaft was embedded in high strength cement. The humeral head was smeared with butter oil and then one-fourth inserted into the high strength cement to mold a partial cup, allowing for an even load to be applied to the humeral head via the cement cup to simulate the glenoid.

**Table 1:** Sociodemographic data and statistical significance between groups

Variable	Non-augmented group (locking plate alone)	Augmented group (plate and strut)	*P*-value
Gender (Male/Female)	3/2	1/4	---
Average age (years)	73.6 (7.4)	77.2 (7.7)	0.472
Average bone mineral density (mg/cm^3^)	247.4 (50.8)	244.4 (36.3)	0.917

Values in bracket are standard deviation

Titanium locking plates were used in this study for constructs fixation. The locking plate was designed from the author’s working group, and United Orthopedic Corporation (United Orthopedic Corporation, Taiwan) manufactured this as a prototype (Unify).

**Fig. 1:**
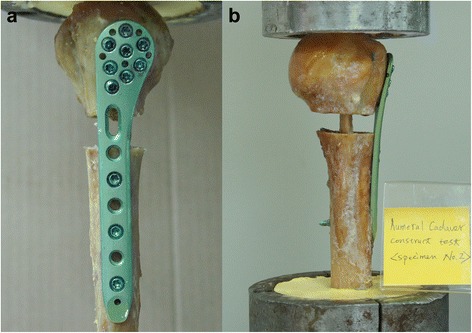
**a** Specimen fixed with a locking plate. Proximal humerus with defect localization and locking plate fixation under compression loads. Three 3.5-mm locking screws were placed into the diaphysis with eight 3.5-mm locking screws into the head. **b** Fixation using a locking plate combined with an intramedullary cortical strut

In the non-augmented group, fractures were fixed with a locking plate alone. Holes were pre-drilled in the near cortex with a 2.8-mm drill bit, and eight 3.5-mm locking screws were placed at the head (Fig. 1a). Five 3.5-mm locking screws were placed through the proximal holes of the plate into the humeral head fragment. Three 3.5-mm locking screws were used for shaft fixation and inserted into the far cortex. In each humerus, a 10-mm gap osteotomy was created at the level of the surgical neck to simulate the comminution commonly encountered with proximal humerus fractures [12, 27]. In the augmented group (locking plate augmented with a cortical bone strut), the same fixation system was augmented (allograft) with a 12-cm long by 1-cm wide cortical bone strut (Fig. 1b). The distal part of the strut was inserted into the canal through the fracture region, and fixed with three distal shaft locking screws which captured the bone strut. The upper part of the strut was retrograded into the humeral head; then the proximal fragment was fixed with eight locking screws through all of the holes at the head of the plate; among them, three screws were inserted through the upper part of the strut to lift the humeral head superiorly.

### Biomechanical testing

Each intact specimen was first installed on the testing system (Instron ElectroPuls E3000, UK) and statically loaded with a displacement rate of 5 mm/min up to 450 N to evaluate the stiffness of intact bone. Each intact specimen was then underwent osteotomy and was fixed with a plate alone or a plate augmented with an intramedullary strut for the subsequent cyclic testing. In the cyclic test, each construct was first preloaded to 10 N and then cyclically axially loaded to 450 N with a sinusoidal waveform at a frequency of 1 Hz, for a total of 6000 cycles. The load and cyclic protocol used in this study aimed to simulate the activities of daily living during early postoperative functional therapy over a period of 6–8 weeks, and to provide more information on the short-term performance of constructs than static loads [28–30].

During the cyclic test, the first 100 preconditioning cycles were applied to obtain more stable readings; therefore the 100th cycle was defined as the first comparative cycle (the initial cycle). The load and displacement data for each cycle was continuously recorded throughout the whole cyclic test. The peak-to-peak (inter-cyclic) displacement and cumulated deformation at specific cycles (100th and every 1000 cycles) were evaluated as the comparison parameters. During the experiments, the specimens were kept moist by spraying with normal saline.

After cycling, each specimen was quasi-statically loaded up to failure at a rate of 5 mm/min. Failure of the construct was evidenced on the load-displacement curve by a sudden drop during cycling, or defined as the maximum applied force or the osteotomy gap of the specimens closed (contact with the fracture site) in the quasi-static test.

### Statistical analysis

The variables used in this study included BMD of the cadaver, displacement of the construct at preselected loading and cycles, and types of fixation (i.e., locking plate alone and plate combined with intramedullary strut). The homogeneity of bone density for the groups was confirmed by one-way analysis of variance (ANOVA). The stiffness and failure strength of each group were presented as mean and standard deviation. Student *t-*tests were used to compare differences between groups. All statistical analyses were performed using Microsoft Excel, and the level of significance between groups was set at *p <* 0.05.

## Results

**Fig. 2:**
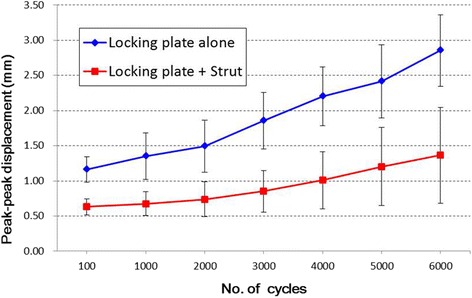
Mean peak-to-peak displacement with the number of cycles under 450 N repeated loading. Curves represent the mean ± SD values for five specimens in each group

During cycling, there was a larger displacement in the non-augmented group compared with the augmented group at the 100th and every 1000th cycle. The mean peak-to-peak (intercycle) displacement increased from 1.2 to 2.9 mm through the cyclic testing for the non-augmented group compared to 0.6 to 1.4 mm in the augmented group. Figure 2 illustrates the peak-to-peak displacements versus cycle curves for the augmented and non-augmented groups under axial cyclic loading. After 6000 cycles the non-augmented group showed a 2 times larger mean peak-to-peak displacement than the augmented group (2.86 ± 0.51 mm vs. 1.36 ± 0.68 mm).

The maximum loads and displacements at 100, 1000, 3000, 6000, and failure cycles for each group were listed in Table 2. All of the specimens in both groups withstood 6000 cycles with 450 N loading without failure. The measured displacements at the 450 N load point (100–6000 cycles) were 1.6 to 3.1 mm for the non-augmented group and 0.7 to 1.7 mm for the augmented group. The displacements were statistically higher in the non-augmented group than in the augmented group (*p* = 0.03). The load to failure was an average of 991 ± 98 N in the non-augmented and 1988 ± 309 N in the augmented group. On average, the maximum load was about two times higher in the augmented group compared to the non-augmented group, and this difference was statistically significant (*p* < 0.001). The measured displacements at failure load were 5.7 mm (range 4.9 to 6.4 mm) for the non-augmented group and 3.5 mm (range 2.1 to 4.2 mm) for the augmented group (*p* = 0.011). The mode of failure for all specimens in both groups was varus collapse combined with loss of screw fixation in the humeral head.

**Table 2:** Maximum load and displacement at specific cycles for each group

Parameter	Non-augmented group	Augmented group	*P*-value
Failure load (N)	991 (98)	1988 (309)	<0.001
Displacement at 100 cycles (mm)	1.6 (0.38)	0.7 (0.26)	0.01
Displacement at 1000 cycles (mm)	1.9 (0.40)	0.9 (0.38)	0.02
Displacement at 3000 cycles (mm)	2.5 (0.58)	1.3 (0.52)	0.02
Displacement at 6000 cycles (mm)	3.1 (0.75)	1.7 (0.65)	0.03
Displacement at failure load (mm)	5.7 (0.72)	3.5 (0.98)	0.01

Mode of failure: varus collapse with loss of screw fixation in all specimens

Values in bracket are standard deviation

## Discussion

Although proximal humeral fractures are often treated with locking plate fixation alone, an unexpectedly high failure rate has been reported when using locking plates in proximal humeral fractures with screw cutout, with failure of fixation typically occurring due to varus deformity or collapse and most frequently in elderly and osteoporotic patients [8, 14, 31, 32]. Studies have reported the stability of the locking and non-locking plates or interlocking intramedullary nails are significantly associated with the BMD [20, 25, 26]. In the current study, the averaged bone densities obtained from the non-augmented and augmented groups were 247.4 ± 50.8 g/cm^3^ and 244.4 ± 36.3 g/cm^3^ (*p* = 0.917), respectively. These two groups can be seen with the equal bone quality and with the osteoporosis. Additionally, the same plate and screw geometry was used in both groups, with the hypothesis that a locking plate augmented with cortical bone strut would be more stable and stronger in an osteoporotic humeral head than in non-augmented constructs. The results showed that the augmented constructs had significantly lower displacement and higher post-cyclic failure strength compared with the non-augmented constructs. This implies that the intramedullary strut shared the applied load and provided internal support to resist axial loading and bending moment due to eccentric load induced by lateral plating, and decreased the stress on the locked screw thereby reducing the potential of varus of the humeral head and screw cutout.

Osteoporotic bone has a weak mechanical structure, and once locking plates are placed on the lateral proximal humerus, the fixed-angular screws behave as cantilever beams to fully support the humeral head fragment to resist varus collapse. As a cyclic varus moment is applied, the repetitive axial loading leads to an impact on cancellous bone by repeated high compression at the tips of the locking screws, gradually cutting into the cancellous bone to form a fan-shaped blade path in the humeral head (subsidence of the screws), resulting in varus deformation. This has been biomechanically confirmed in previous studies [20, 28], and may explain the loss of reduction, screw perforation or cut-out in osteoporotic bone observed under cyclic loading. The failure mode for all specimens were varus collapse combined with loss of screw fixation in the humeral head. Our results were consistent with the clinical outcome and previous biomechanical studies [8–24].

The reaction force about the shoulder joint at 90° of isometric abduction has been biomechanically evaluated to be 0.9 to 1.4 times the body weight [33]. Praagman et al. reported the maximum compressive force through the shoulder at 90° of elevation to be about 400 N across the gleno-humeral joint [34]. Laursen et al. also reported a less than 500 N maximum push force across the gleno-humeral joint [35]. Anglin evaluated the average contact forces ranged from 1.3 (using the arms to stand up from and sit down into a chair) to 2.4 times body weight when lifting a 10 kg suitcase [36]. Zettl et al. compared the deformation under 450 N load for two locking plate/screw system with respect to biomechanical stability [29]. The quasi-static test showed the non-augmented and augmented groups failed at 991 and 1988 N axial loads, respectively, which were both greater than 1.3 times a body weight of 75 kg (975 N). Both groups were capable of sustaining the loads of arms to push body stand up from a chair. Even though the augmented groups could provide the capacity of 2.4 times body weight (to lift up a 10 kg suitcase), we do not suggest over 450 N loading in the post-operative phase.

Although fibular allografts have been used in a clinical setting [12, 23], we used cortical bone struts harvested from the cortex of the diaphysis of the humerus to emphasize the importance of intramedullary struts in unstable proximal humeral fractures with poor bone quality. In the current study, the plates were designed with 14 locking holes and one compression hole. However, our constructs used eight locking screws into the head and three into the diaphysis for fixation. It is unclear whether filling every screw hole in the plate will decrease the rate of cutout, however, it can be reasonably assumed that more screws will result in stronger fixation. Although the locked angular screw behaves as a cantilever beam to press the cancellous bone, micro-damage forms in the cancellous bone and then enlarge the migration of the humeral head with a plate alone, once the screw inserted into an intramedullary strut, the strut provides a support at the tip of the screw to decrease the subsidence. In our augmented constructs, both the proximal and distal parts of the strut were fixed with three locking screws (three at the head and three at the shaft of the humerus), which not only secured the strut but also provided at least three additional cortices of screw purchase thereby preventing loosening of the implant or screw pullout from the humeral head. We think that a plate-screw-strut is a more rigid construct to stabilize the fixation.

It is difficult to compare our results with those obtained in other investigations due to the use of different fracture patterns, loading conditions, experimental set-up and types of implant. Although the locking plates and screws used in this study are not commercially available, the aim of the experiment was to biomechanically investigate the role and importance of intramedullary cortical bone struts, and to compare the mechanical properties of proximal humeral fractures treated with or without cortical struts in the same type of plate. Fibular grafts have been used to treat proximal humerus and humeral shaft fractures, and the results have shown improved nonunion rates. However, geometrical size-matching problems between the fibula and medullary canals have been shown to exist. In the case of a humerus with a narrow canal, the canal has to be enlarged by reamers to allow for insertion of an appropriately contoured fibula. The intramedullary struts in our study were harvested from the humeral shaft and trimmed to a width of 1-cm and a length of 12-cm; clinically, therefore, the strut can be customized to fit a patient’s humeral canal. In our specimens, it was difficult to maintain a consistent distance between the plate and strut because of variations in the diameter of the canal of the shaft. Placement of an intramedullary strut near the medial canal may increase the lever arm of the strut from the plate, thereby providing more resistance to a varus moment. Although positioning the intramedullary strut near the medial canal can decrease varus deformation of the humeral head, based on the results of our study, we suggest that an intramedullary strut can provide sufficient initial stability and strength to withstand 450 N of axial load, even if it is not positioned at the medial canal.

Our constructs can be compared to Brianza et al’s novel fixation technique, in which they combined expert proximal humeral nails with a special locking plate to improve the interfragmentary stability [28]. Their device provided medial column support which significantly decreased varus displacement of the articular fragment under axial compression. A recently developed locking plate was combined with a helical blade to achieve local bone compaction providing additional bone purchase and an increased stability of the calcar region [37]. The additional insertion of an inferomedially placed helical blade significantly reduced the occurrence of secondary varus displacement. Similarly, our constructs combined a locking plate and an intramedullary strut to stabilize the fixation, and the strut also provided medial column support.

Although, the effect of the length of the cortical strut was not investigated in our study, we used a 12-cm long cortical bone strut which was longer than the plate (11-cm long), a recent study reported no differences in interfragmentary motion with struts of different lengths [38]. Therefore, the length of the intramedullary struts in our study seems to be sufficient.

There are several limitations to this study. First, although the homogeneity of bone density of the specimen was statistically matched between groups as possible, inter-individual differences among cadavers do exist and may lead to variations in the results. Second, each specimen was stripped of all soft tissues, and thus the stability provided by surrounding soft tissues was not evaluated. Third, the fracture patterns and screw configurations may have affected the stability of the constructs. In this study, an osteotomy gap was used to represent a fracture, and thus our experimental model may not reflect complex 3 or 4-part fracture patterns. Furthermore, in the dynamic testing the complete sequence of arm motion could not be simulated. Thus, the results are only conditionally transferable to the in vivo situation. Other limitations are that the number of specimens was small, and that the loading protocol was also limited.

## Conclusions

This study investigated the cyclic stability and failure strength of unstable proximal humeral fractures fixed using locking plate together with an intramedullary cortical bone strut. We conclude that the locking plate combined with an intramedullary cortical bone strut could provide about two times of mechanical stability and strength for constructs using locking plate alone. The strut provided a medial column support to create a medial column support to reduce the varus moment of the humeral head and reduced migration and the amount of cumulated deformation. The stability and strength of the augmented constructs might be sufficient to allow the upper extremities to be used for unloaded abduction or simple activities of daily living.

## Abbreviations

ANOVA: Analysis of variance
BMD: Bone mineral density
CT: Computed tomography
